# Genome-wide analysis of *14-3-3* gene family in four gramineae and its response to mycorrhizal symbiosis in maize

**DOI:** 10.3389/fpls.2023.1117879

**Published:** 2023-02-17

**Authors:** Yanping Wang, Qiang Xu, Hanchen Shan, Ying Ni, Minyan Xu, Yunjian Xu, Beijiu Cheng, Xiaoyu Li

**Affiliations:** ^1^ National Engineering Laboratory of Crop Stress Resistance Breeding, Anhui Agricultural University, Hefei, China; ^2^ Yunnan Key Laboratory of Plant Reproductive Adaptation and Evolutionary Ecology and Institute of Biodiversity, School of Ecology and Environmental Science, Yunnan University, Kunming, China

**Keywords:** gramineous, *14-3-3*, maize, evolution, AM symbiosis

## Abstract

*14-3-3* proteins (regulatory protein family) are phosphate serine-binding proteins. A number of transcription factors and signaling proteins have been shown to bind to the *14-3-3* protein in plants, which plays a role in regulating their growth (seed dormancy, cell elongation and division, vegetative and reproduction growth and stress response (salt stress, drought stress, cold stress). Therefore, the *14-3-3* genes are crucial in controlling how plants respond to stress and develop. However, little is known about the function of *14-3-3* gene families in gramineae. In this study, 49 *14-3-3* genes were identified from four gramineae, including maize, rice, sorghum and brachypodium, and their phylogeny, structure, collinearity and expression patterns of these genes were systematically analyzed. Genome synchronization analysis showed large-scale replication events of *14-3-3* genes in these gramineae plants. Moreover, gene expression revealed that the *14-3-3* genes respond to biotic and abiotic stresses differently in different tissues. Upon arbuscular mycorrhizal (AM) symbiosis, the expression level of *14-3-3* genes in maize significantly increased, suggesting the important role of *14-3-3* genes in maize-AM symbiosis. Our results provide a better understanding on the occurrence of *14-3-3* genes in Gramineae plants, and several important candidate genes were found for futher study on AMF symbiotic regulation in maize.

## Introduction

The 14-3-3 protein was originally isolated from bovine brain tissues by Moore and Perez (Carlson and Hubbard, 1968). The *14-3-3* family is composed of several subunits, exists in all eukaryotes, such as yeast (Heusden et al., 2010), human (Boston and Jackson, 1980) and Arabidopsis thaliana (Rosenquist et al., 2001). In *Arabidopsis thaliana*, they major components of the protein/G-box complex, and they were named “G-box factor 14-3-3” or “GF14” (Lu et al., 1992). 14-3-3 proteins (regulatory protein family) are phosphate serine-binding proteins that regulate the activity of a series of targets through direct protein-protein interactions (Bridges and Moorhead, 2005). A number of transcription factors and signaling proteins have been shown to bind to the 14-3-3 protein in plants, which plays a role in regulating their growth [seed dormancy (Darling and Yingling, 2005), cell elongation and division (Zhang et al., 2010), vegetative and reproduction growth (Asih et al., 2009; John, 2012)] and stress response [salt stress (Wei et al., 2009), drought stress (Chevalier et al., 2009), cold stress (Yao et al., 2007)].

Arbuscular mycorrhizal (AM) fungi belong to the ancient spherical phylum and are soil-borne microorganisms. They can establish the most extensive symbiotic relationship with around 80% of terrestrial flowering plant species, i.e. AM symbiosis (Simon et al., 1993; Remy et al., 1994). AM fungi consume plant photosynthates (Bago et al., 2000) and lipids for the duration of their life cycle (Bravo et al., 2017; Jiang et al., 2017). In return, AM fungi not only enhance the absorption of mineral nutrients and water for host plants, but also protect them from fungal pathogens (Jung et al., 2012; Chitarra et al., 2016) and all kinds of abiotic stresses (for example, heavy metals, low available nutrients, drought, extreme temperatures, acidic soils, salinity, aluminum toxins and contaminants) (Augé, 2001; Schützendübel and Polle, 2002; Lenoir et al., 2016). Therefore, AM fungi are the key endophytes of plant symbiosis. Symbiosis has an important impact on plant productivity and ecosystem functions (Van der Heijden and Klironomos, 1998), and is critical to agricultural sustainability (Gianinazzi et al., 2010).

At present, the AM fungal induced functions of *14-3-3* gene are mainly related to drought resistance. For example, *Gi14-3-3* gene is involved in the protection of AM symbiosis against drought stress in host plants (Porcel et al., 2006). AM symbiosis decreases the expression of two plasma membrane aquaporin genes (PIP) activated by ABA signal, which reduces cell membrane permeability and improves cell water retention (Porcel et al., 2006). D-inositol-3-phosphate synthase (IPS) and 14-3-3 protein GF14 (14-3GF) are also regulated by AM fungi to activate cross-talk between symbiotic partners and increase the drought resilience of maize plants (Li et al., 2016). AM symbiosis may regulate stomatal behavior and maintain water use efficiency by regulating *14-3-3* genes in ABA signal pathway, thus improving drought resistance of plants (Xu et al., 2018). Moreover, it has also been reported that *14-3-3* genes can promote symbiosis. For example, three 14-3-3 protein genes have been identified in AMF: Fm201, Ri14-3-3, and RiBMH2. their transcriptional levels are highly induced in the early stage of symbiosis and symbiotic stage, including germinating spores, stem hyphae and roots (Sun et al., 2018).

Therefore, understanding the evolutionary relationship of *14-3-3* of Gramineae and the induction and regulation of *14-3-3* genes by AM symbiosis is of great significance to improve the response of crops to biotic and abiotic stresses

## Materials and methods

### Identification of the 14-3-3 proteins in maize, sorghum, rice, and brachypodium

The genome and predicted protein sequences for maize (*Zea mays*), rice (*Oryza sativa-IRGSP-1.0*), sorghum (*Sorghum bicolor*), and brachypodium (*Brachypodium distachyon-v3.0*) were obtained from the respective databases: MaizeGDB (http://www.maizegdb.org/, accessed on 20 October 2022), Phytozome (http://www.phytozome.net/ accessed on 20 October 2022), and Ensembl Plants (http://plants.ensembl.org, accessed on 20 October 2022). The version number for four plant databases are Zea mays-v4, Oryza sativa Japonica Group-IRGSP-1.0, Sorghum bicolor-v3, Brachypodium distachyon-v3. Two techniques were used to identify the gene encoding 14-3-3 from four plants at the whole genome level. Initially, functionally known 14-3-3 protein sequences from Arabidopsis (AtGRF1) and Oryza sativa (OsGF14a) were employed to query homologous proteins ofmaize, rice, sorghum, and brachypodium using BLASTp with E-values of less than le^-5^ (Huang et al., 2015). Then, Pfam scan (http://www.ebi.ac.uk/Tools/pfa/pfamscan/, accessed on 20 October 2022) was used to validate the existence of a 14-3-3-related domain (PF00244.23) in all 14-3-3 protein sequences.

### Protein sequence analysis, chromosomal locations, gene structure and protein motif identification analysis

The theoretical isoelectric point (pI) and molecular weight (MW, kDa) were calculated using the ProtParam tool in ExPASy (http://web.expasy.org/protparam/, accessed on 22 October 2022) (Elisabeth et al., 2003)). Each protein’s subcellular localization was predicted using the Cell-PLoc 2.0 server (http://www.csbio.sjtu.edu.cn/bioinf/Cell-PLoc-2/, accessed on 22 October 2022). The ensemblplant Database (http://plants.ensembl.org/index.html, accessed on 22 October 2022) was used to find the chromosomal locations of the *14-3-3* genes. The exon/intron structures of the *14-3-3* genes were determined using the Gene Structure Display Server (GSDS 2.0: http://gsds.cbi.pku.edu.cn/ (Hu et al., 2014), accessed on 22 October 2022) The Multiple Expectation Maximization for Motif Elicitation program (MEME, http://alternate.meme-suite.org, accessed on 22 October 2022) was used to identify the conserved motif of 14-3-3 proteins in four gramineae, the parameter was set as any number of repetitions and the maximum value of motif was 15 to identify the conserved motif of 14-3-3 protein in four gramineae plants (Bailey et al., 2006).

### Duplication analysis

Evolutionary selection is measured by the ratio of non-synonym to synonymous substitution (Ka/Ks). In the TBtools software (v1.098726) (Chen et al., 2020), the genome databases and gene annotation files of four species were analyzed by using the MCScanX function, and the homologous gene pairs were brought into the Ka/Ks function for calculation, thus the evolutionary relationship was obtained. Homologous gene pairs can also be used for collinear analysis to evaluate the assembly effect of genomes and the retention and loss of homologous genes by homologous comparison, and to study the evolutionary relationship of materials. We integrated GFF file format files, gene link format files and Chr Layout format files using File Merge for MCScanX package on TBtools (v1.098726). Then the synteny relationships of *14-3-3* genes in four species were constructed.

### Gene expression profile

Gramene database (http://www.gramene.org/, accessed on 1 November 2022) was utilized to gather tissue expression data. We selected the expression data of the required species from the website and downloaded it to search for the required *14-3-3* gene family genes. Then we selected the heatmap function in Tbtools (v1.098726) and imported the organized expression pattern data into it and click star. The *14-3-3* gene expression data of four gramineous plants were investigated and heat maps were drawn.

### Plant materials, growth conditions, and treatments

Maize B73 seeds were germinated in hydroponic culture under sterile conditions. After germination, we moved seedlings of maize B73 to a sand-based pot culture to inoculate them with arbuscular mycorrhizal fungus. The control group was not inoculated with arbuscular mycorrhizal fungi. Culture medium was mixed with vermiculite:perlite at a 4:1 ratio and sterilised by 40 min high-pressure steam at 121℃. The AMF species was Glomus intraradices (Gi, provided by Sun Yat-Sen University). We grew maize at 28°C with 16 hours of light and 8 hours of darkness in a greenhouse. After 60 days post-treatment, the plants were collected. For RNA isolation, samples were stored at 80°C.

TransZol Up Plus RNA Kit was used to extract total RNA (TransGen Biotech, Beijing, China). cDNAs were obtained using reverse transcriptase (Vazyme, Nanjing, China). *ZmTubulin* (Zm00001d009780) and *ZmGAPDH* (Zm00001d049641) were used as the endogenous reference genes to standardize the relative expression levels of *ZmGRF* genes. Our previous published work provided extensive knowledge regarding qRT-PCR reactions (Wang et al., 2022). The 10^-(ΔCt/3)^method was used to determine the relative gene expression levels (Li et al., 2005; Liao et al., 2014). The qPCR assays were performed with three biological replicates. 

### Construction of regulatory network of symbiosis-related *ZmGRFs*

According to the qRT-PCR results, the co-expression network of *14-3-3* genes induced by AMF with significant differences was constructed. First, the *ZmGRFs* co-expression data was downloaded from PlantRegMap (http://plantregmap.gao-lab.org/, accessed on 2 November 2022), a plant transcriptional regulation map database. Secondly, we measured the RNAseq data of maize symbiosis [PRJNA918441(SAMN32395817-SAMN32395900)]. Co-expression networks were created using the WGCNA package (v1.63) in R with a soft threshold of 18 (Langfelder and Horvath, 2008). Expression modules were obtained using the automatic network construction function block wise Modules with default settings. The modules were grouped using a stringency threshold of 0.75. The two regulatory networks were intersected to obtain the primary expression regulatory network. The co-expression network was visualized using the Cytoscape program (v3.6.1) (Shannon et al., 2003).

## Results

### Identification of *14-3-3* gene family in maize, sorghum, rice, and brachypodium

In the current study, 49 genes were identified as members of *14-3-3* family in the genome of four plants. Maize *14-3-3* gene family was named according to the reported naming methods of Arabidopsis *14-3-3* gene family. This study found 28 *ZmGRF* genes in the maize genome, 6 in the sorghum genome, 7 in the brachypodium genome, and 8 in the rice genome. The number of 14-3-3 genes in maize is about four times that of other three species. It is speculated that corn needs 14-3-3 to play different functions to adapt to the environment. These *14-3-3s* were designated as *ZmGRF*, *SbGF14*, *OsGF14*, and *BdGF14* followed by number or alphabet. The detailed information of *14-3-3* genes were listed in **Table 1**, **Tables S1–S3**, including chromosome distribution, gene length, isoelectric point, and molecular weight. The number of amino acid residues in the four plants 14-3-3 proteins ranged from 94 (ZmGRF27) to 501 (ZmGRF3), and their relative molecular weights (MWs) ranged from 10.87 kDa (ZmGRF27) to 53.42 kDa (ZmGRF3). Theoretical pI (isoelectric point) values ranged from 4.71 (OsGF14e 、 OsGF14g 、 BdGF14b) to 9.69 (ZmGRF26) and rice, sorghum and brachypodium were all less than 7. Subcellular localization prediction of *14-3-3* gene family showed that only *ZmGRF4* was located in Cytoplasm and *ZmGRF27*、*ZmGRF28* 、*OsGF14g*、 *OsGF14h*、 *SbGF14f*、 *BdGF14g* were located in cytoplasm and Nucleus, while the other 42 14-3-3 proteins were all located in Nucleus. The chromosomal location maps revealed that *ZmGRFs* were found on nine of ten chromosomes, whereas *SbGF14s* were found on three of ten chromosomes (Chr. 5,6, and 7), and *OsGF14s* were mainly existed on six of twelve chromosomes (Chr.1, 2, 3, 4, 8, and 11), and *BdGF14s* were dispersed on four of five chromosomes (Chr.1, 3, 4, and 5).

### Phylogenetic and structural analysis of 14-3-3s in maize, sorghum, rice, and Brachypodium

**Figure 1:**
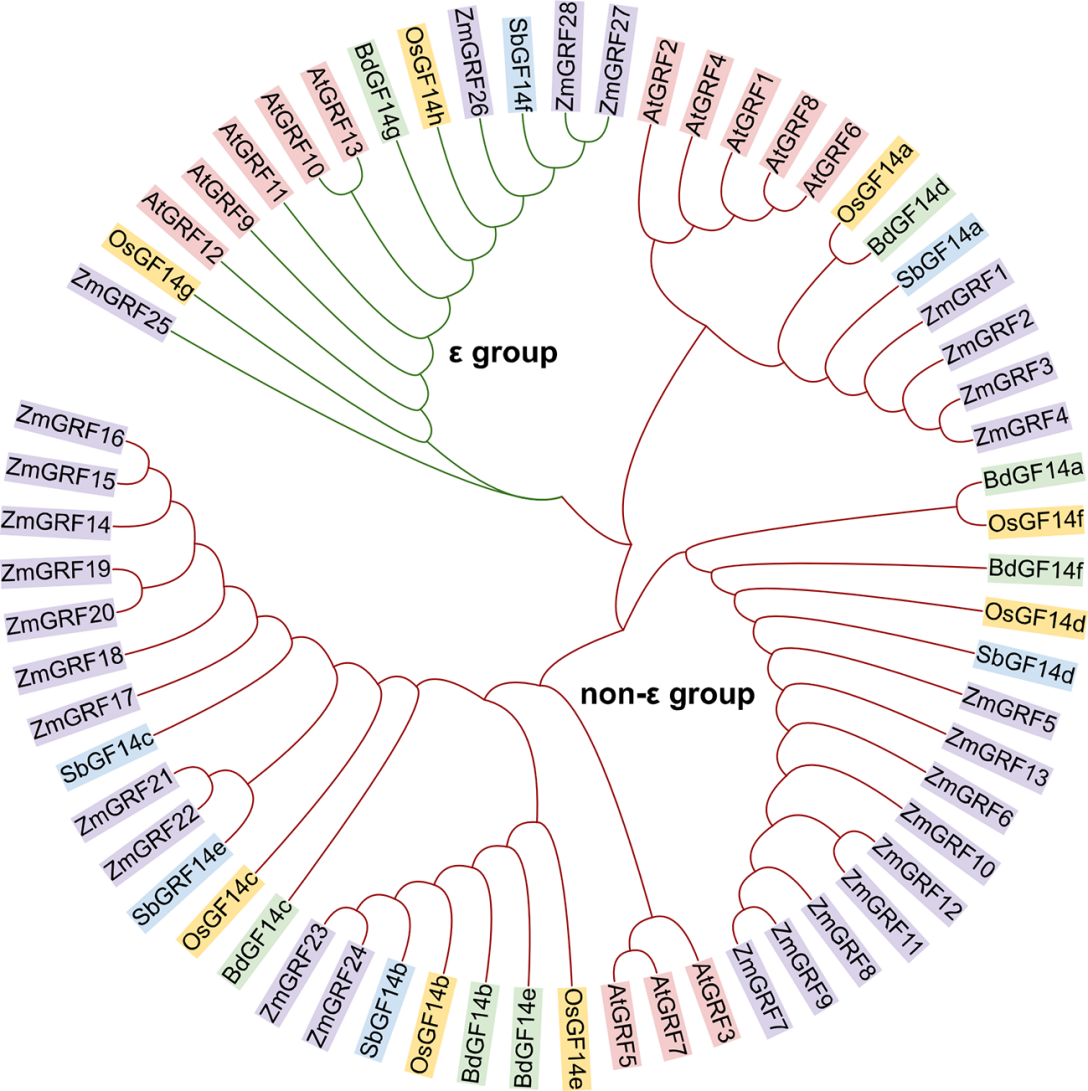
Phylogenetic relationship among 14-3-3 proteins in plants. The 14-3-3 protein sequences of Arabidopsis thaliana, maize, rice, sorghum and Brachypodium were mapped using *Multiple Sequence Alignment by CLUSTALW*. The 14-3-3 proteins in the phylogenetic tree were divided into two groups (ϵ group and non-ϵ classes). At, *Arabidopsis thaliana*; Zm, *Zea mays*; Os, *Oryza sativa*; Sb, *Sorghum bicolor*; Bd, *Brachypodium distachyon*.

*14-3-3* genes from maize, sorghum, rice, and brachypodium were studied for evolutionary relationships. Firstly, the 14-3-3 proteins of four species and the reported 14-3-3 proteins of *Arabidopsis thaliana* were analyzed by phylogenetic tree. According to the group of *Arabidopsis thaliana*, the 14-3-3s of five plants were divided into ϵ and non-ϵ group (**Figure 1**). The phylogenetic differentiation patterns of the five plants were similar, but the four gramineae were more closely related than arabidopsis, reflecting different 14-3-3 conserved evolution and function. Among the four gramineae species, ϵ-group contains 8 members, accounting for 16.3% of the total 14-3-3s. Non ϵ-group contains 41 members, accounting for 81.7% of the total 14-3-3s. In ϵ group, ZmGRF25 has a distant genetic relationship with other genes, which may be caused by evolutionary selection to adapt to the environment. In non- ϵ classes, it can be seen from the evolutionary tree that there are three branches. Each branch can be regarded as an ancestor’s 14-3-3 gene. Through replication and species differentiation, each branch represents gene pedigree-specific replication and loss. This is of instructive significance for us to judge the evolution and origin of 14-3-3 gene family in Gramineae.

**Figure 2:**
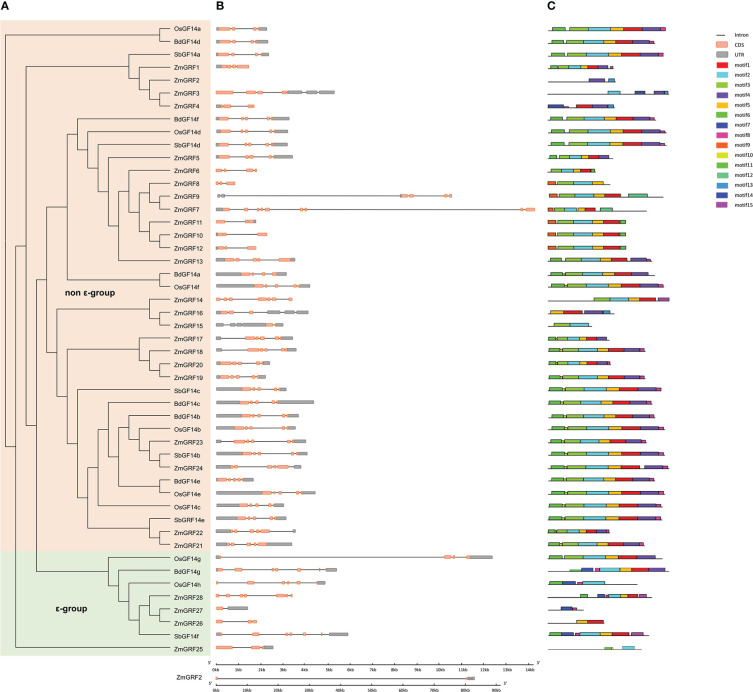
Phylogenetic tree, motif, and gene structure of maize, sorghum, brachypodium, and rice. **(A)**. The phylogenetic tree of four plants. non - ϵ group and ϵ group indicate the two *14-3-3* subfamilies. *14-3-3s* from maize (*ZmGRF1* to *ZmGRF28*), sorghum (*SbGF14a* to *SbGF14f*), brachypodium (*BdGF14a* to *Bd GF14g*), and rice (*OsGF14a* to *OsGF14h*). **(B)**. Gene structure of four plants. Exons are indicated by pink boxes, introns are indicated by single lines, and gray boxes represent untranslated regions (UTR); **(C)**. Motifs’ analysis. Different colors represent different motifs.

To further understand the structural diversity of *14-3-3* genes and the diversity of protein structure, The exon/intron structure of *14-3-3* genes and the conserved motif of protein sequences were compared (**Figure 2**). Highly conserved distribution of CDS regions (Almost all the *14-3-3s* contained 3 to 5 in numbers, except *ZmGRF7*, *ZmGRF14*, *ZmGRF15*, *ZmGRF27*, *ZmGRF28*, *OsGF14h*, *SbGF14f* and *BdGF14g*) was found among *14-3-3* genes. To further understand the structural variety of 14-3-3 proteins, online MEME/MAST tools were used to analyze the conserved motifs. The MEME program was used to predict fifteen putative protein motifs. In non - ϵ group and ϵ group, the motifs were not regular, but they all contained at least one of the first 8 motifs. In ϵ group, except ZmGRF26, other seven genes all contained motif7 and motif8. In non - ϵ group, all 14-3-3 proteins contained motif1, motif2, Motif3 and Motif5 except ZmGRF2, ZmGRF3, ZmGRF4, ZmGRF8, ZmGRF15 and ZmGRF16. Motif sequences were shown in **Table S4**.

### Duplication events of *14-3-3* genes among maize, sorghum, rice, and brachypodium

**Figure 3:**
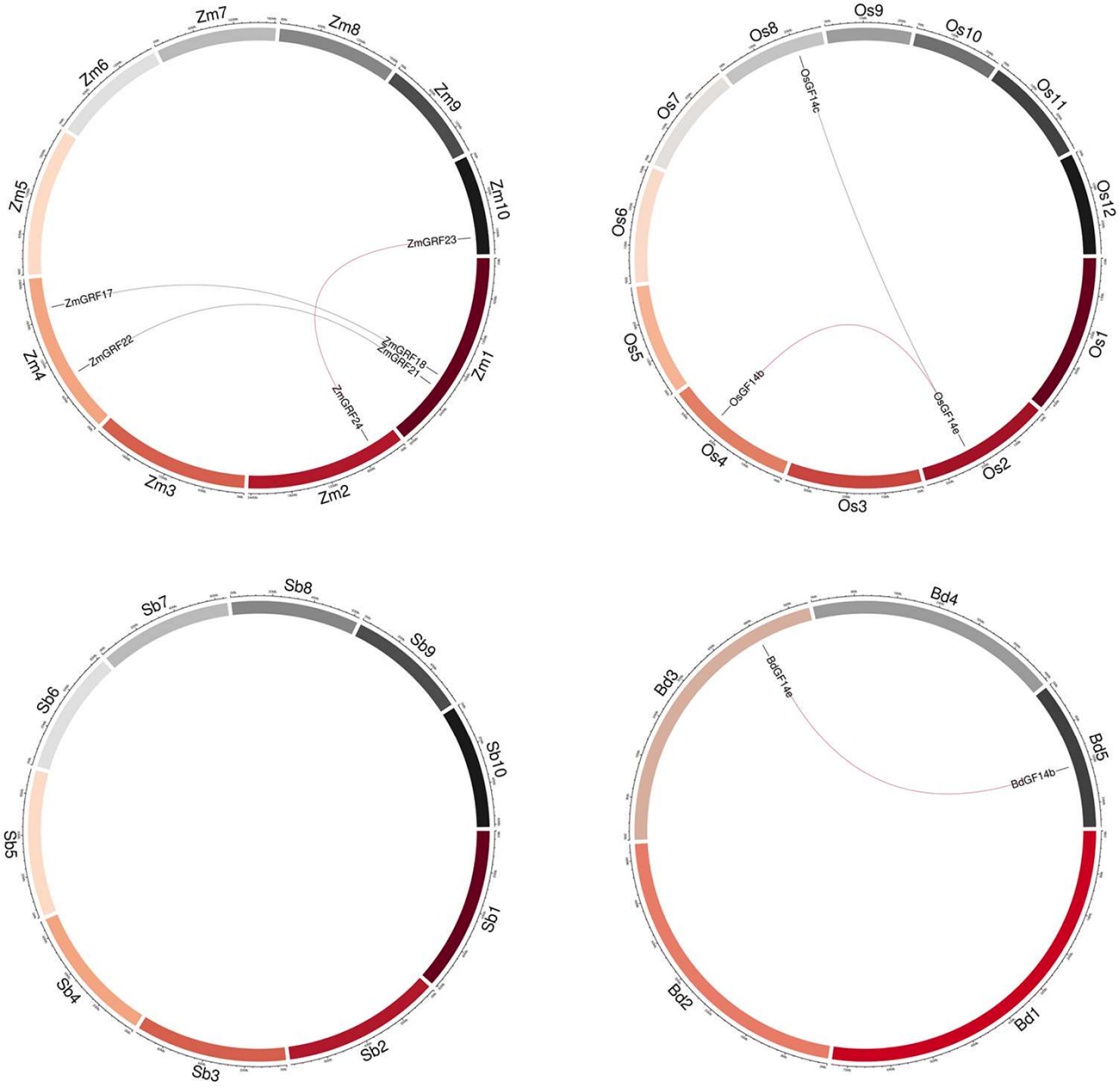
Synteny of four plants *14-3-3* genes. Zm1–10 represented maize chromosome 1–10, Sb1–10 represented sorghum chromosome, Bd1–5 represented brachypodium chromosome and Os1–12 represented rice chromosome, all chromosome indicated by colored boxes. Chromosome box numbers represent sequence lengths in megabases. All the syntenic genes were located in the map.

According to the whole-genome analysis in the four gramineae plants, maize had the most putative duplicated gene pairs (*ZmGRF17*/*ZmGRF18*, *ZmGRF21*/*ZmGRF22*, and *ZmGRF23*/*ZmGRF24*), while rice contained two pairs (*OsGF14b*/*OsGF14e*, *OsGF14c*/*OsGF14e*), brachypodium contained one pair (*BdGF14b*/*BdGF14e*), and sorghum had no gene pairs (**Figure 3**).

**Figure 4:**
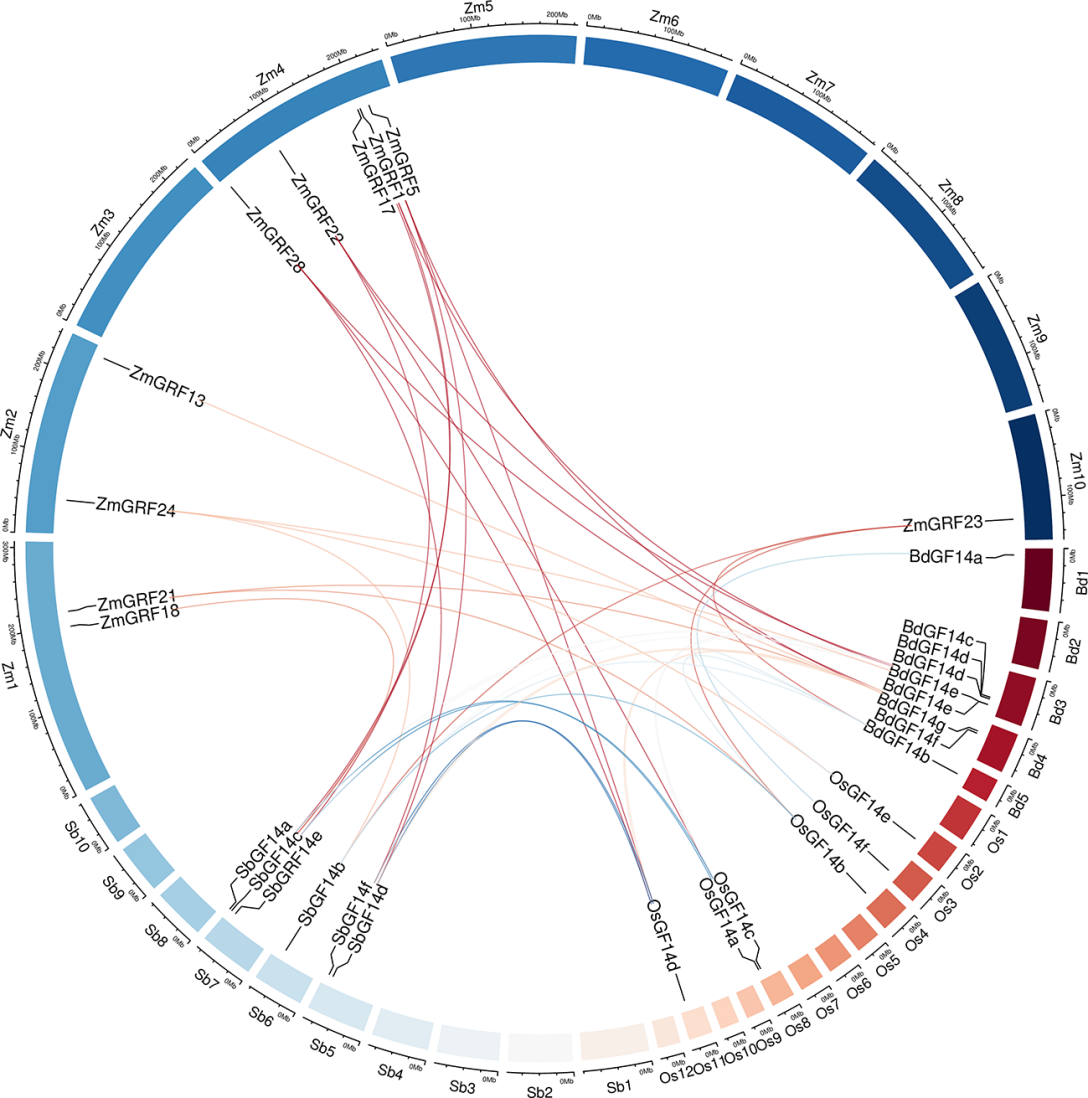
Circle plot showing segmentally duplicated *14-3-3* genes on maize, sorghum, brachypodium, and rice chromosomes. Zm1–10 represented maize chromosome 1–10, Sb1–10 represented sorghum chromosome, Bd1–5 represented brachypodium chromosome and Os1–12 represented rice chromosome, all chromosome indicated by colored boxes. Chromosome box numbers represent sequence lengths in megabases. lines indicated duplicated *14-3-3* gene pairs.

For better comprehending of these genes, we explored the gene duplication events of them in four Gramineae plants. To better understand the evolution history of the *14-3-3* gene family, putative orthologous links between all the *14-3-3* genes in maize and other three plants were established (**Figure 4**, **Table 2**).

We identified 6 homologous gene pairs between maize and rice (*ZmGRF5* and *OsGF14d*;*ZmGRF21* and *OsGF14b*; *ZmGRF22* and *OsGF14c*; *ZmGRF23* and *OsGF14b*; *ZmGRF24* and *OsGF14e*; *ZmGRF28* and *OsGF14h*), and 8 homologous gene pairs between maize and sorghum (*ZmGRF1* and *SbGF14a*; *ZmGRF5* and *SbGF14d* ; *ZmGRF17* and *SbGF14c*; *ZmGRF18* and *SbGF14c*; *ZmGRF22* and *SbGF14e*; *ZmGRF23* and *SbGF14b*; *ZmGRF24* and *SbGF14b*; *ZmGRF28* and *SbGF14f*), 8 homologous gene pairs between maize and brachypodium (*ZmGRF1* and *BdGF14d* ; *ZmGRF5* and *BdGF14f* ; *ZmGRF13* and *BdGF14f*; *ZmGRF21* and *BdGF14c*;ZmGRF22 and *BdGF14c*; *ZmGRF23* and *BdGF14b* ;*ZmGRF24* and *BdGF14e*; *ZmGRF28* and *BdGF14g*) (**Figure 4**). The difference number of orthologous gene pairs between maize and the other three plants may be due to different loss rates of duplicated genes during the evolutionary processes. Except for 10 genes in the above gene pairs, other *ZmGRFs* did not appear in any duplication block in maize. *OsGF14g* of rice also did not appear in any duplication block.

The ratio of nonsynonymous-to-synonymous substitutions (Ka/Ks) was used to calculate evolutionary selection. When the ratio of a pair of sequences Ka/Ks is less than one, purifying selection is inferred; when it is equal to one, neutral drift is inferred; and when it is greater than one, positive or Darwinian selection is inferred (Juretic, 2005; Chen et al., 2014). In four gramineae plants, the selection analysis revealed that duplicated gene pairs were mostly subjected to purifying selection (Ka/Ks < 1.0). (**Table 2**).

### Tissue specificity expression of *14-3-3s* in maize sorghum, rice, and brachypodium

**Figure 5:**
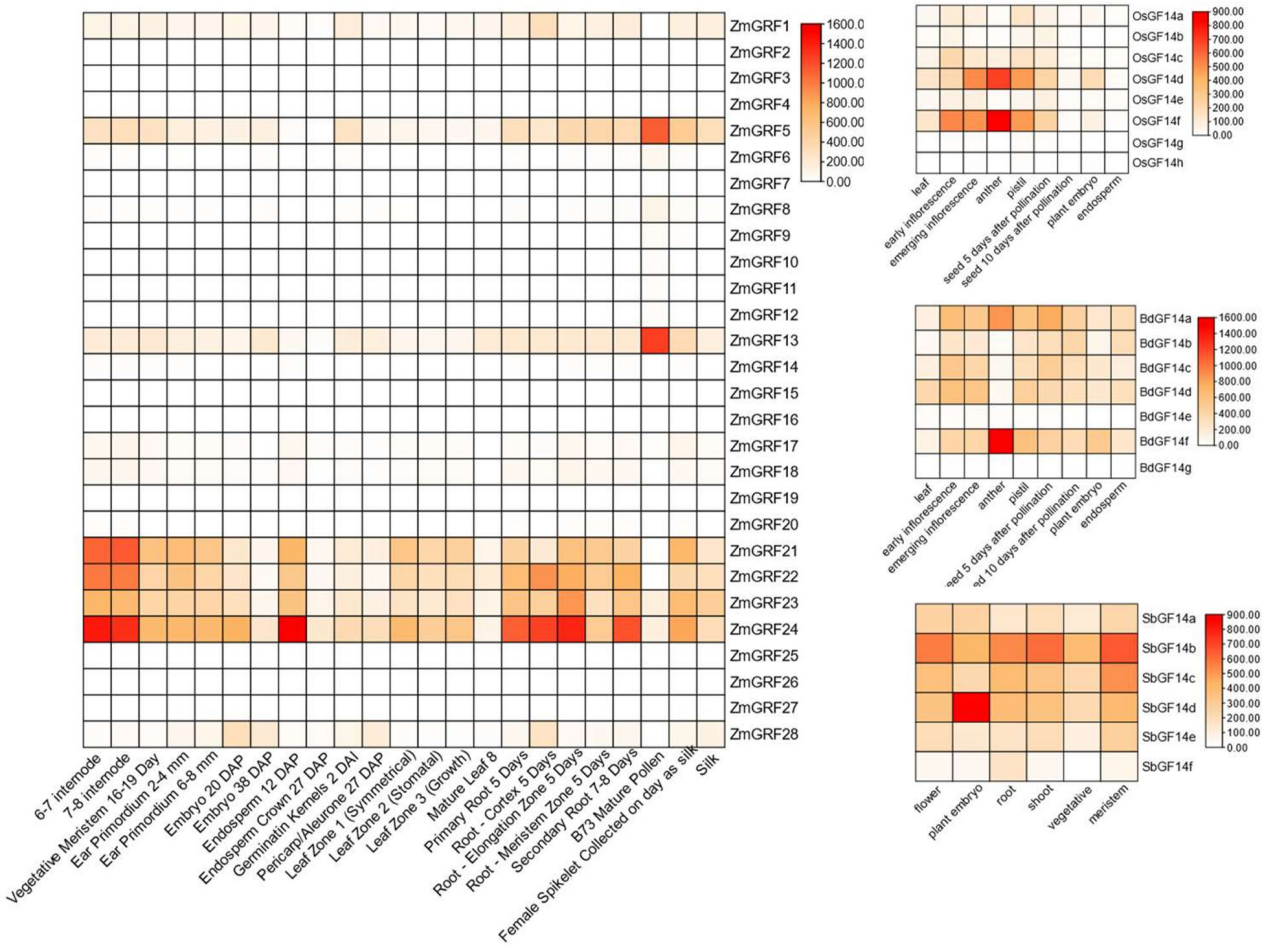
Heat maps of maize, sorghum, brachypodium, and rice *14-3-3* genes. Color scale at the right represents log_2_-transformed foldchange values. Red indicates high expression level; yellow indicates medium expression level; and white indicates low expression level.

With reported data, the expression levels of the *14-3-3* genes in four gramineae plants from different tissues at different times were used to create a heat map (**Figure 5**) (Davidson et al., 2012; Sekhon et al., 2013). According to the heat map, *ZmGRF1*、*ZmGRF5*、*ZmGRF13*、*ZmGRF21*、*ZmGRF22*、*ZmGRF23*、*ZmGRF24* and *ZmGRF28* were expressed in all tissues. *ZmGRF5* and *ZmGRF13* were highly expressed in pollen, which might be closely related to reproductive development; *ZmGRF22*、*ZmGRF23* and *ZmGRF28* were highly expressed in internodes, which might be related to water and nutrient transport; In addition, *ZmGRF28* was also highly expressed in 12 days immature embryos and roots at all stages, which was speculated to be related to reproduction and nutrient transport. Other *ZmGRFs* were low or not expressed in all tissues. *OsGF14d*, *OsGF14f* and *BdGF14f* were highly expressed in anthers in rice and brachypodium, which might be closely related to reproductive development; Other genes were moderately or low expressed in the tissues shown by the heat map. In sorghum, all genes were expressed in all tissues, with *SbGF14b* being significantly expressed in flowers, stems, and meristems; *SbGF14d* was highly expressed in embryos. But the repeat pairs of maize, rice and Brachypodium showed different expression patterns, suggesting that 14-3-3s have different functions in maize, rice and Brachypodium.

### Expression patterns of *ZmGRF* genes in mycorrhizal roots

**Figure 6:**
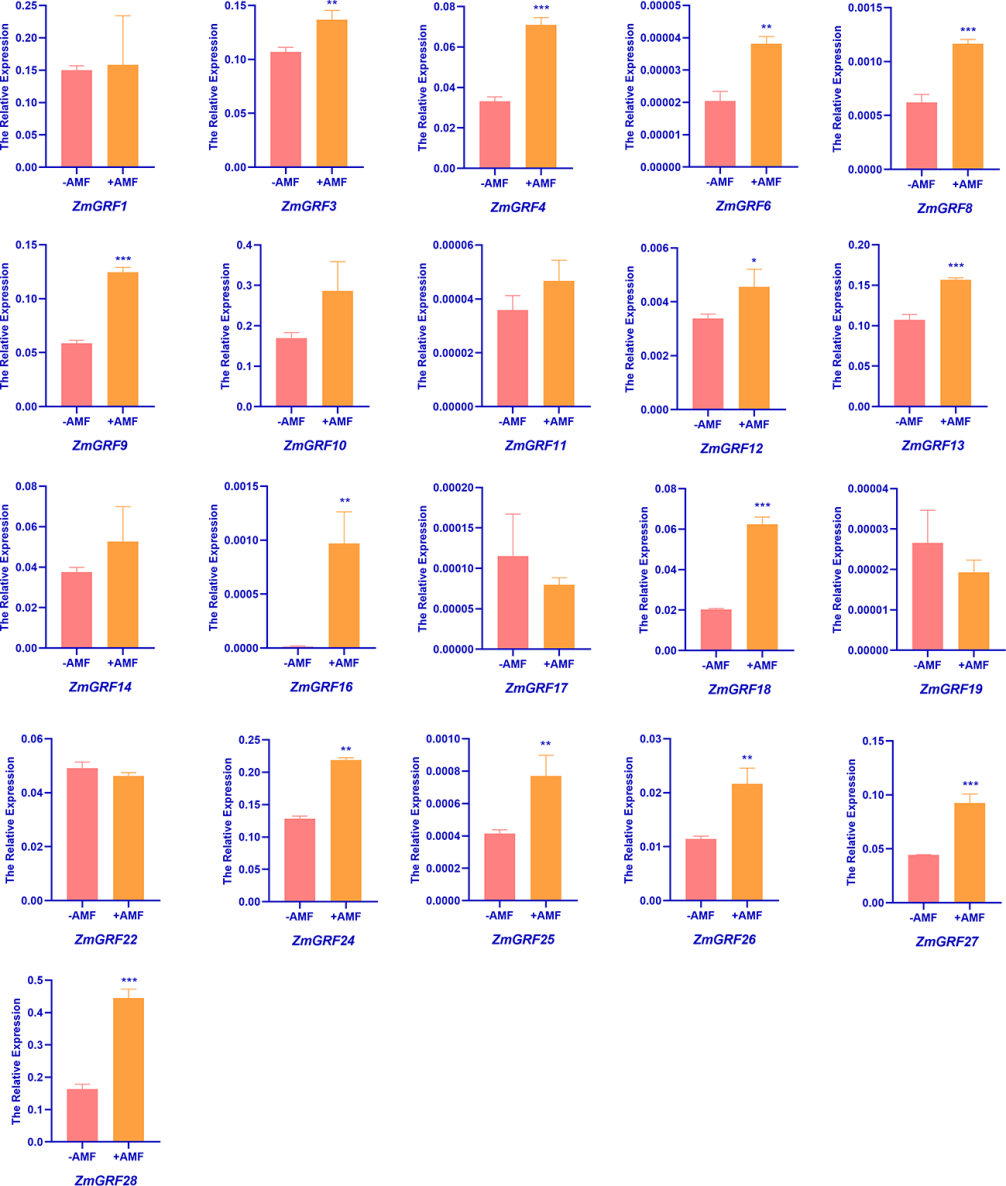
Relative expressions of *14-3-3* genes in roots of wild-type. Maize was inoculated with AM symbiosis and sampled 60 days after inoculation. (p*<0.05、p**<0.01、p***<0.001).

In order to understand the expression of *14-3-3* genes induced by AM fungi in maize, RT-qPCR was performed using cDNA of maize roots inoculated with and without AM fungi as templates. We totally found 28 sequences of *ZmGRF* genes, but only 21 of genes were identified by qRT-PCR, while *ZmGRF2*, *ZmGRF5*, *ZmGRF7*, *ZmGRF15*, *ZmGRF20*, *ZmGRF21* and *ZmGRF23* could not be identified in maize. The expression level of the 21 *ZmGRF* genes were analyzed by RT-PCR (**Figure 6**). The primer pairs are listed in **Table S5**. Compared with non-AM symbiosis, there was no significant difference in the expression of *ZmGRF1*, *ZmGRF10*, *ZmGRF11*, *ZmGRF14*, *ZmGRF17*, *ZmGRF19* and *ZmGRF22* genes after AM symbiosis, indicating that these six genes were not induced by AM symbiosis. The expression of *ZmGRF12* gene was significantly up-regulated (P < 0.05); The expression of *ZmGRF3*, *ZmGRF6*, *ZmGRF16*, *ZmGRF24*, *ZmGRF25* and *ZmGRF26* genes were significantly up-regulated (P < 0.01), The expression of *ZmGRF4*, *ZmGRF8*, *ZmGRF9*, *ZmGRF13*, *ZmGRF18*, *ZmGRF27* and *ZmGRF28* genes were significantly up-regulated (P < 0.001), indicating that these 14 genes were up-regulated by AM symbiosis. It was speculated that these genes might had a significant impact in the symbiotic pathway, and further research is needed to determine the specific mechanism.

**Figure 7:**
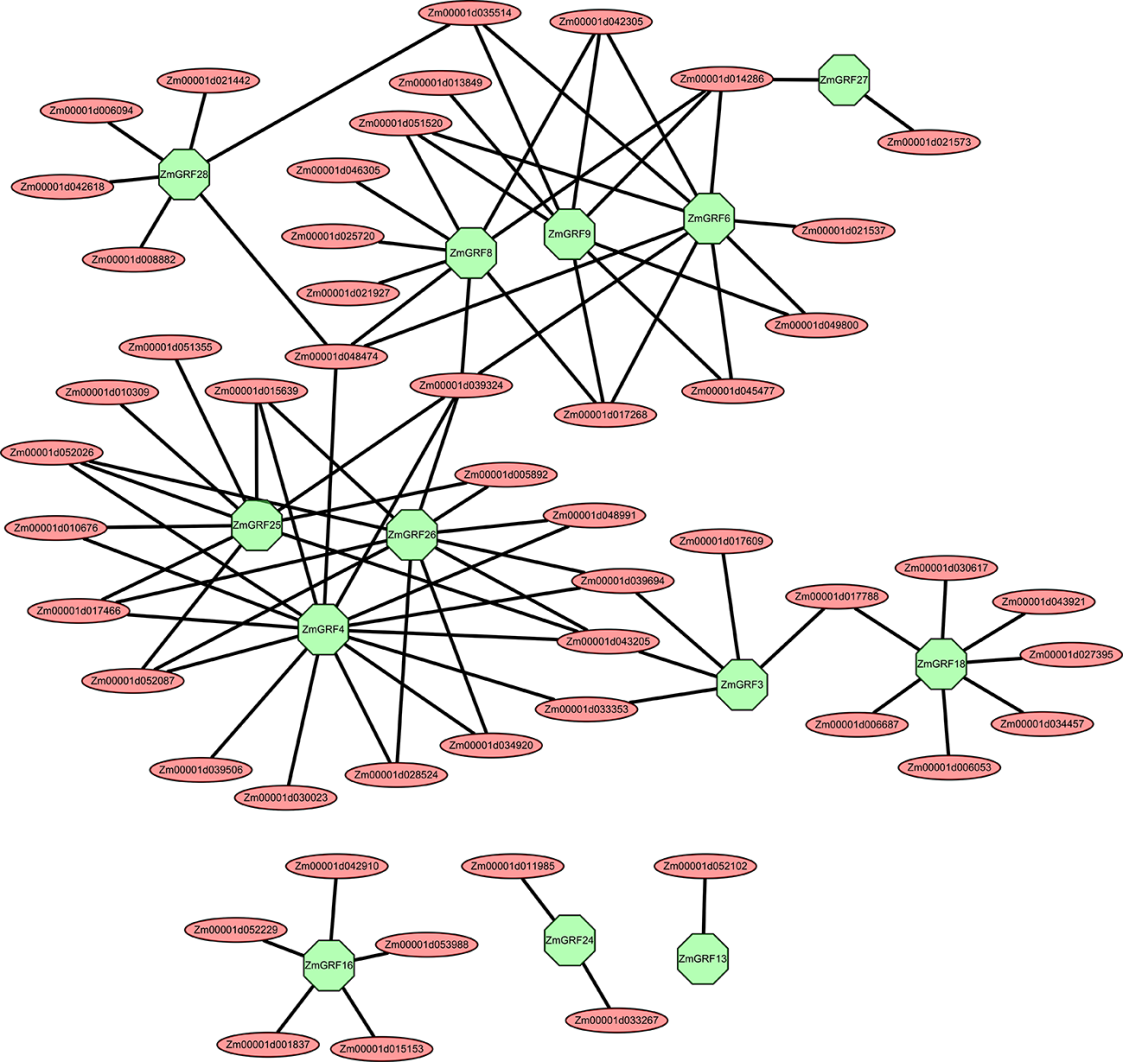
The transcriptional regulatory networks of 13 symbiosis-related maize *14-3-3s*. These transcriptional regulatory networks were constructed by Cytoscape software. Red represents regulatory genes, and green represents symbiosis-related genes in the *14-3-3* gene family.

To further verify that the above genes could be induced by AM fungi, the promoter cis-acting elements of the above 13 *14-3-3* genes were analyzed (**Figure 7**). W-BOX (TTGACC), OSEROOTNODULE (AAAGAT) (JLQ et al., 2008), CTTC element (TCTTGT) (Lota et al., 2013), were cis-acting elements associated with AM symbiosis, and NODCON2GM (CTCTT) was a cis-acting element associated with root nodule symbiosis. The cis-acting element map was drawn for the 2000 bp upstream promoter through the RSAT website (http://rsat.eead.csic.es/plants/dna-pattern_form.cgi, accessed on 25 October 2022). It can be seen from the figure that the promoters all contain the above four cis-acting elements.

### Interaction and regulatory networks prediction of symbiosis-related *ZmGRFs*

Transcriptional regulatory networks of TFs are particularly important for identifying target regulatory genes and interacting proteins of TFs (Barabási and Oltvai, 2004; Jang et al., 2015). It is still largely unknown how *14-3-3s* regulate the graminaceous - AM symbiosis, particularly in maize. In this study, we selected 13 symbiotic-related maize *14-3-3* genes based on the results of RT-qPCR and constructed their co-expression networks through the plant transcriptional regulation map database and symbiotic-related RNA-seq data. According to the predicted regulatory network, *ZmGRF4*, *ZmGRF6*, *ZmGRF25* and *ZmGRF26* had relatively more regulatory genes, about 10-15. *ZmGRF 3*, *ZmGRF 8*, *ZmGRF 9*, *ZmGRF 16*, *ZmGRF 18* and *ZmGRF 28* were followed by about 5-9 regulated genes. *ZmGRF13*, *ZmGRF 24* and *ZmGRF 27* were the least, with only 1-2 regulated genes (**Table S6** and **Figure 7**). Among the predicted regulatory genes, the following five transcription factor gene families account for most of them, namely Ethylene-responsive transcription factor, *AP2/EREBP* transcription factor, *MADS* transcription factor, *MYB* transcription factor and *NAC* transcription factor. These transcription factor families had been reported in plant-AM symbiosis. For example, the Medicago *AP2/EREBP* transcription factor *WRI5a* plays a role in controlling bidirectional nutrient exchange and periarbuscular membrane formation by regulating genes involved in fatty acid biosynthesis and phosphate uptake in arbuscular cells (Jiang et al., 2018).*MYB1* regulated the transcriptional program of arbuscular degeneration during AM symbiosis (Floss et al., 2017).Expression of *MtNAC969* in symbiotic root nodules was induced by nitrate treatment and antagonized by salt in roots and nodules, similar to markers of senescence (Zélicourt et al., 2012).The above results indicated that AM symbiosis was a very complex process involving multiple regulatory pathways and exerting different functions during the symbiosis process.

## Discussion

14-3-3 protein have been identified and studied in many species (Cheng et al., 2018; Wang et al., 2019; Liu et al., 2020), but its evolutionary origin and phylogenetic relationship have not been fully clarified. In this study, there are 49 genes in the *14-3-3* gene family found in four plants. The number of *14-3-3* genes in maize is about 4 times that of the other three species, which may be in the process of evolution, and the function of the *14-3-3* genes is very different, which is particularly important for plant environment adaptation. Moreover, we further analyze the properties (amino acid length, molecular weight and isoelectric point) of 14-3-3 proteins (**Table 1**, **Tables S1**-**S3**). Physicochemical properties of 14-3-3 protein shows that the proteins of the other three species were acidic and stable, consistent with previous reports. But ZmGRFs proteins are partially acidic and partially alkaline, which is similar to Pe14-3-3s (Guo et al., 2022).Similarly, the diversity of gene structure can provide more in-depth insights for the study of structure, evolution and functional relationships (Xu et al., 2012). Except maize, the other three species all contain more than 4 introns. This indicates that evolution may lead to the diversity of this structure. Phylogenetic analysis shows that 14-3-3 proteins in four gramineae are divided into ϵ and non-ϵ groups, which is consistent with previous reports in other species (**Figure 1** and **Figure 2**) (Rosenquist et al., 2001; Wei, 2006; Wang et al., 2019).14-3-3 proteins are highly conserved in eukaryotes (Aitken et al., 1993) and can form homologous and heterodimers (Jones et al., 1995). They can interact with two different target proteins to form complexes at the same time (Yaffe et al., 1997; Ottmann et al., 2007; Taoka et al., 2011). The analysis of conserved motifs show that most of the *14-3-3* gene families of four gramineae have 8 conserved motifs, which are the core structures that bind with many ligands (Yang et al., 2006).

**Table 1:** Information of 14-3-3s in maize.

Gene name	Gene ID	Chr.	Exon number	CDS(bp)	Protein length(aa)	Theoretical pI	Mw (KDa)	Subcellular localization
*ZmGRF1*	Zm00001d052796_T003	4	4	810	270	4.86	29.71	Nucleus.
*ZmGRF2*	Zm00001d015779_T001	5	5	537	179	7.02	19.92	Nucleus.
*ZmGRF3*	Zm00001d039374_T001	3	7	1,503	501	9.27	53.42	Nucleus.
*ZmGRF4*	Zm00001d028091_T001	1	4	531	177	8.8	19.35	Cytoplasm.
*ZmGRF5*	Zm00001d053090_T001	4	5	807	269	4.8	29.54	Nucleus.
*ZmGRF6*	Zm00001d048554_T001	9	4	594	198	8.24	22.09	Nucleus.
*ZmGRF7*	Zm00001d039042_T001	6	9	1,224	408	9.41	45.46	Nucleus.
*ZmGRF8*	Zm00001d021885_T001	7	3	498	166	9.13	18.02	Nucleus.
*ZmGRF9*	Zm00001d018856_T001	7	7	909	303	8.97	33.67	Nucleus.
*ZmGRF10*	Zm00001d033357_T001	1	3	630	210	8.84	23.3	Nucleus.
*ZmGRF11*	Zm00001d007968_T001	2	3	630	210	8.84	23.36	Nucleus.
*ZmGRF12*	Zm00001d017434_T001	5	3	630	210	8.68	23.3	Nucleus.
*ZmGRF13*	Zm00001d007446_T003	2	5	831	277	4.8	30.55	Nucleus.
*ZmGRF14*	Zm00001d034593_T001	1	7	966	322	5.81	35.9	Nucleus.
*ZmGRF15*	Zm00001d013429_T001	5	4	351	117	5.13	13.63	Nucleus.
*ZmGRF16*	Zm00001d006462_T001	2	6	450	150	4.9	17.16	Nucleus.
*ZmGRF17*	Zm00001d052698_T001	4	6	759	253	4.77	28.59	Nucleus.
*ZmGRF18*	Zm00001d031688_T001	1	6	774	258	4.81	29	Nucleus.
*ZmGRF19*	Zm00001d036226_T001	6	5	774	258	4.76	29.03	Nucleus.
*ZmGRF20*	Zm00001d038649_T002	6	5	765	255	4.81	29.07	Nucleus.
*ZmGRF21*	Zm00001d032231_T001	1	5	771	257	4.82	28.95	Nucleus.
*ZmGRF22*	Zm00001d050375_T001	4	6	771	257	4.78	28.86	Nucleus.
*ZmGRF23*	Zm00001d025617_T001	10	6	786	262	4.75	29.63	Nucleus.
*ZmGRF24*	Zm00001d003401_T003	2	6	819	273	4.72	30.39	Nucleus.
*ZmGRF25*	Zm00001d050903_T001	4	3	1,149	383	8.91	41.98	Nucleus.
*ZmGRF26*	Zm00001d035316_T001	6	3	450	150	9.69	17.29	Cytoplasm.
*ZmGRF27*	Zm00001d036341_T001	6	2	282	94	6.73	10.87	Cytoplasm. Nucleus.
*ZmGRF28*	Zm00001d048868_T002	4	8	1,290	430	8.58	47.39	Cytoplasm. Nucleus.

The expansion of plant gene family has experienced whole-genome replication and tandem replication, which has led to plant evolution and created diversity of gene functions (Bowers et al., 2003; Doyle et al., 2008). The difference between the four gramineae plants may be caused by the whole-genome replication event (Sun et al., 2017). It can be seen from the results, a total of 6 fragment repetition events were identified in the other three plants except sorghum (**Figure 3**), supporting this hypothesis. We also performed a collinear analysis of the four species (**Figure 4**), guessing that they originated from the same ancestor. Therefore, genes may have similar functions. The Ka/Ks ratio can be used to indicate the direction of gene selection (Cannon et al., 2004; Shiu, 2004; Sun et al., 2017). In this study, 14-3-3 genes are subjected to purifying selection (**Table 2**), and it is speculated that there was little difference in function (Hurst, 2002). These results provide reference for the study of 14-3-3 genes.

**Table 2:** Evolutionary selection between *14-3-3s* in maize and other three plants.

Seq 1	Seq 2	*Ka*	*Ks*	*Ka/Ks*
*ZmGRF1*	*BdGF14d*	0.058011353	0.463964254	0.125034099
*ZmGRF1*	*SbGF14a*	0.023365238	0.292741213	0.079815336
*ZmGRF5*	*BdGF14f*	0.040664956	0.500546013	0.081241195
*ZmGRF5*	*SbGF14d*	0.008319553	0.213613171	0.038946816
*ZmGRF5*	*OsGF14d*	0.051048859	0.532322412	0.095898384
*ZmGRF13*	*BdGF14f*	0.037232687	0.488049001	0.076288829
*ZmGRF17*	*SbGF14c*	0.040080774	0.189114488	0.211939204
*ZmGRF17*	*ZmGRF18*	0.045421666	0.23182265	0.195932823
*ZmGRF18*	*SbGF14c*	0.032352386	0.244809988	0.132153046
*ZmGRF21*	*BdGF14c*	0.042931003	0.57891128	0.074158173
*ZmGRF21*	*OsGF14b*	0.087701953	1.087064238	0.080677801
*ZmGRF21*	*ZmGRF22*	0.015376393	0.222394573	0.069140144
*ZmGRF22*	*BdGF14c*	0.037598234	0.627674635	0.059900833
*ZmGRF22*	*OsGF14c*	0.025258294	0.944399938	0.026745337
*ZmGRF22*	*SbGF14e*	0.006805661	0.275950022	0.024662659
*ZmGRF23*	*BdGF14b*	0.022007504	0.639569901	0.034409849
*ZmGRF23*	*OsGF14b*	0.015149905	0.656259945	0.02308522
*ZmGRF23*	*SbGF14b*	0.001667826	0.151035206	0.011042628
*ZmGRF23*	*ZmGRF24*	0.004185279	0.201407049	0.0207802
*ZmGRF24*	*BdGF14e*	0.041112047	0.8828826	0.046565701
*ZmGRF24*	*OsGF14e*	0.038992073	1.147124688	0.033991138
*ZmGRF24*	*SbGF14b*	0.001672008	0.183559219	0.009108823
*ZmGRF28*	*BdGF14g*	0.205725142	0.617297626	0.333267347
*ZmGRF28*	*OsGF14h*	0.117418878	0.493725337	0.237822265
*ZmGRF28*	*SbGF14f*	0.021004873	0.142640598	0.147257329

The expression patterns of 14-3-3 genes are different in different tissues of different plants (Li et al., 2016). In this study, *14-3-3* genes of the four plant species show different expressions in tissues (**Figure 5**), indicating that they play different roles in plant responses to different biological processes. The same has been reported in other plants (Cheng et al., 2018; Xu et al., 2021; Xia et al., 2022). For example, *ZmGRF5*, *ZmGRF13*, *OsGF14d*, *OsGF14f* and *BdGF14f* are highly expressed in anthers in rice and brachypodium, which may be closely related to reproductive development. It has been previously reported that *AtGRF12* is highly expressed in flowers and floral organs (Tian et al., 2015). These results indicate that tissue expression pattern provides a reference direction for studying gene function.

**Figure 8:**
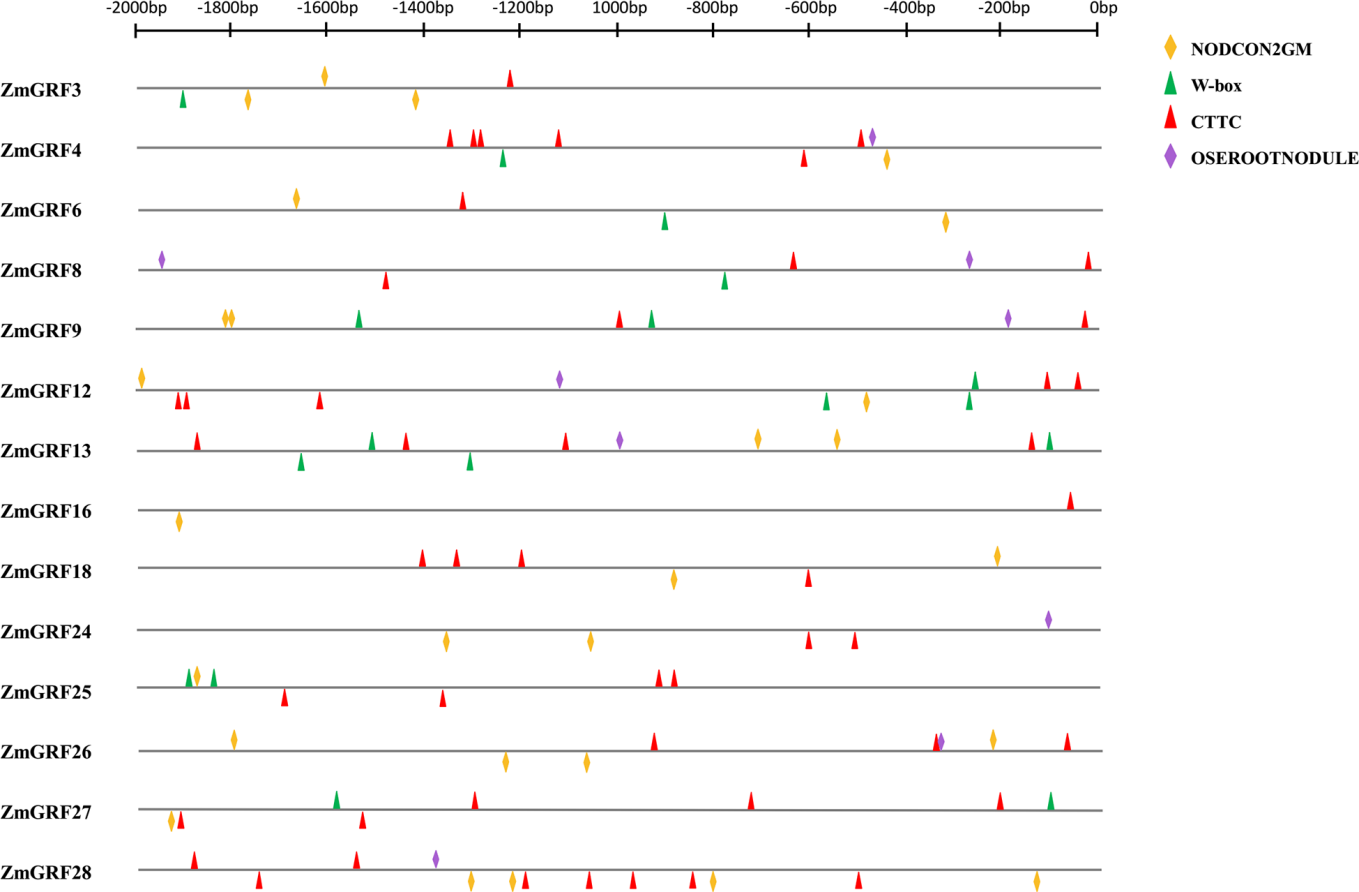
Promoter cis-acting element analysis. The promoter fragments are 2.0 kb in length. The yellow block represents the NODCON2GM motif. The green block represents the W-box motif. The red block represents the CTTC motif. The purple block represents the OSEROOTNODULE motif.

Gramineae is an important food crop. People are very interested in AM fungal symbiosis to improve crop resistance. It has previously been reported that the expression of *14-3-3* genes induced by AM fungi can improve drought tolerance in plants (Porcel et al., 2006; Porcel et al., 2006; Li et al., 2016). In addition, the overexpression of *Fm201*, *Ri14-3-3*, and *RiBMH2* genes can promote the symbiosis between plants and AM fungi (Xu et al., 2018). The results show that 21 *ZmGRFs* genes were significantly up-regulated after induction by AM fungi, while the remaining 7 *ZmGRFs* are not expressed or had no significant differences (**Figure 6**). Next, the cis-acting elements of the 13 *14-3-3* gene promoters are analyzed, and it is found that they all containe elements related to AM fungal induction (**Figure 8**) (JLQ et al., 2008; Lota et al., 2013). These results further confirm that maize *14-3-3* gene family plays an important role in AM fungal symbiosis, but the detailed mechanism remains to be studied.

Different proteins interact to form complexes to perform various biological functions (Morsy et al., 2008; Braun et al., 2013). A co-expression network is constructed for 13 AM fungi-induced *14-3-3* genes. Three of the maize *14-3-3* genes have interacting proteins that differ from the other ten members, according to the predicted interacting proteins. The three genes are *ZmGRF13*, *ZmGRF16* and *ZmGRF24* respectively (**Figure 7**). It is speculated that the difference may be caused by the need to participate in other response pathways in order to adapt to environmental changes during evolution. Among the regulated transcription factors, there are 30 *AP2/EREBP* transcription factor family genes, 10 *MYB* transcription factor family genes, 7 *MADS* transcription factor family genes, 5 *NAC* transcription factor family genes, and the number of other transcription factor family genes is less than 5 (**Table S6**). These transcription factor families have been reported in other species to play different roles in the symbiotic process of AM fungi. This co expression network has certain guiding significance for studying the mechanism of transcription factor regulating *14-3-3* genes in the symbiotic process.

In conclusion, Plant 14-3-3 proteins can interact with proteins of various biological processes to participate in many key processes. For example, related proteins such as growth and development, material transport and biological and microbial stress. At present, the related factors have been isolated, and the molecular mechanism of 14-3-3 protein has been deeply studied (Aitken, 2006; Toker et al., 2010; Kumar et al., 2015; Camoni et al., 2018). For example, 14-3-3 proteins play a vital function in regulating stomatal movement by regulating binding partners in guard cells (Cotelle and Leonhardt, 2016). By binding to other proteins,14-3-3 proteins regulate the plant’s internal physiological processes in response to changing external environmental conditions (Denison et al., 2011). For example, overexpression of 14-3-3 protein in Arabidopsis thaliana enhances cold tolerance (Visconti et al., 2019). The 14-3-3 proteins are curcial for the response to biological stress (Yang et al., 2009). We aim to study the mechanism of improving plant tolerance to biotic and abiotic stresses by altering the expression pattern of *14-3-3* genes through AM symbiosis. This study identified 14 maize *14-3-3* genes that were up-regulated by AM symbiosis. Maize is an important food crop and cash crop. This study is of great significance to the breeding of excellent maize varieties.

## Conclusion

14-3-3 proteins are involved in a variety of metabolic activities of plants. Although great progresses have been made in the function analysis of *14-3-3* genes in some plants, there are few studies on the 14-3-3 gene family in gramineae. According to the study, we identified 49 *14-3-3* genes in maize, rice, sorghum, and brachypodium and demonstrated that the Gramineae *14-3-3* genes underwent large-scale replication and purifying selection. The expression analysis of *14-3-3* genes revelated that 14-3-3 genes in maize, sorghum, rice and short brachypodium may have different functions. Other species have reported that *14-3-3* genes are induced by AM fungi in response to variety of abiotic stresses. These studies provide basic information for *14-3-3* genes.In this study, the *14-3-3* gene in maize was induced by AM fungi, and 13 genes up-regulated by AM fungi were identified. Promoter analysis and co-expression network analysis were performed for them, which has certain guiding significance for studying the mechanism of transcription factors regulating *14-3-3* gene in response to various biotic and abiotic stresses during symbiosis.

## Data availability statement

The data presented in the study are deposited in the NCBI repository, accession number PRJNA9184 (SAMN32395817-SAMN32395900).

## Author contributions

The authors have made the following declaration about their contributions. Conceived and designed the experiments: YW, QX and HS. Performed the experiments: YW and QX. Analyzed and interpreted the data: HS, YX and YN. Performed statistical analysis: MX. Drafted the manuscript: YW, QX, HS, YN and MX. Edited and finalized the manuscript: BC and YX. All authors contributed to the article and approved the submitted version.
