# Supplementary material for: Genome-wide analysis of 14-3-3 gene family in four gramineae and its response to mycorrhizal symbiosis in maize
Source: Front Plant Sci. 2023 Feb 17;14:1117879. doi: 10.3389/fpls.2023.1117879 (PMC9982033; doi:10.3389/fpls.2023.1117879)
Supplement: Supplementary file 1 [file DataSheet_1.docx]

Supplementary Material

Genome-Wide Analysis of *14-3-3* Gene Family in Four Gramineae and Its Response to Mycorrhizal Symbiosis in Maize

Yanping Wang^1^, Qiang Xu^1^, Hanchen Shan^1^, Ying Ni^1^, Minyan Xu^1^, Yunjian Xu^2^, Beijiu Cheng^1^*, Xiaoyu li^1^*

*** Correspondence:** Beijiu Cheng: cbj@ahau.edu.cn; Xiaoyu Li: lixiaoyu@ahau.edu.cn

**Supplementary Table 1.** Information of 14-3-3s in sorghum.

| **Gene name** | **Gene ID** | **Chr.** | **Exon number** | **CDS(bp)** | **Protein length(aa)** | **Theoretical pI** | **Mw (KDa)** | **Subcellular localization** |
| --- | --- | --- | --- | --- | --- | --- | --- | --- |
| *SbGF14a* | EES14338 | 7 | 4 | 777 | 259 | 4.84 | 28.61 | Nucleus. |
| *SbGF14b* | EES10954 | 6 | 5 | 783 | 261 | 4.76 | 29.65 | Nucleus. |
| *SbGF14c* | EES15274 | 7 | 5 | 759 | 253 | 4.8 | 28.67 | Nucleus. |
| *SbGF14d* | OQU83613 | 5 | 5 | 798 | 266 | 4.8 | 29.34 | Nucleus. |
| *SbGRF14e* | OQU80487 | 7 | 5 | 768 | 256 | 4.79 | 28.93 | Nucleus. |
| *SbGF14f* | EES10091 | 5 | 7 | 741 | 247 | 5.17 | 27.95 | Cytoplasm. Nucleus. |

**Supplementary Table 2.** Information of 14-3-3s in rice.

| **Gene name** | **Gene ID** | **Chr.** | **Exon number** | **CDS(bp)** | **Protein length(aa)** | **Theoretical pI** | **Mw (KDa)** | **Subcellular localization** |
| --- | --- | --- | --- | --- | --- | --- | --- | --- |
| *OsGF14a* | Os08t0480800-01 | 8 | 4 | 792 | 264 | 4.85 | 29 | Nucleus. |
| *OsGF14b* | Os04t0462500-01 | 4 | 5 | 786 | 262 | 4.76 | 29.86 | Nucleus. |
| *OsGF14c* | Os08t0430500-01 | 8 | 5 | 768 | 256 | 4.78 | 28.83 | Nucleus. |
| *OsGF14d* | Os11t0546900-01 | 11 | 5 | 795 | 265 | 4.83 | 29.26 | Nucleus. |
| *OsGF14e* | Os02t0580300-01 | 2 | 7 | 786 | 262 | 4.71 | 29.69 | Nucleus. |
| *OsGF14f* | Os03t0710800-01 | 3 | 6 | 780 | 260 | 4.81 | 29.18 | Nucleus. |
| *OsGF14g* | Os01t0209200-01 | 1 | 7 | 771 | 257 | 4.71 | 28.8 | Cytoplasm. Nucleus. |
| *OsGF14h* | Os11t0609600-01 | 11 | 6 | 600 | 200 | 5.76 | 23.06 | Cytoplasm. Nucleus. |

**Supplementary Table 3.** Information of 14-3-3s in brachypodium.

| **Gene name** | **Gene ID** | **Chr.** | **Exon number** | **CDS(bp)** | **Protein length(aa)** | **Theoretical pI** | **Mw (KDa)** | **Subcellular localization** |
| --- | --- | --- | --- | --- | --- | --- | --- | --- |
| *BdGF14a* | KQK13608 | 1 | 4 | 789 | 263 | 5.02 | 29.59 | Nucleus. |
| *BdGF14b* | KQJ82997 | 5 | 5 | 786 | 262 | 4.71 | 29.68 | Nucleus. |
| *BdGF14c* | KQJ98364 | 3 | 5 | 768 | 256 | 4.79 | 28.81 | Nucleus. |
| *BdGF14d* | KQJ98723 | 3 | 4 | 786 | 262 | 4.8 | 28.91 | Nucleus. |
| *BdGF14e* | KQK00042 | 3 | 5 | 786 | 262 | 4.84 | 29.6 | Nucleus. |
| *BdGF14f* | KQJ88264 | 4 | 5 | 798 | 266 | 4.8 | 29.37 | Nucleus. |
| *BdGF14g* | KQJ87863 | 4 | 7 | 894 | 298 | 6.26 | 33.48 | Cytoplasm. Nucleus. |

**Supplementary Table 4.** Primers of sequences

| **Gene name** | **Gene ID** | **Forward primer** | **Reverse primer** |
| --- | --- | --- | --- |
| *ZmGRF1* | Zm00001d052796_T003 | TACCTCGCCGAGTTCAAGAC | GCGAGCTGAGGATCTCGTAG |
| *ZmGRF2* | Zm00001d015779_T001 | GACGCTGGGCATAATGAAAC | GCCTGGTCTTCGTCTCTGTC |
| *ZmGRF3* | Zm00001d039374_T001 | GAGCTGCTTCTAGGGCTTTGG | ATCCTGTACTGGTCCCGCATT |
| *ZmGRF4* | Zm00001d028091_T001 | CCAAGAATCCCATCAAGCTC | TCCACAAGGTGAGGTTGTCA |
| *ZmGRF5* | Zm00001d053090_T001 | GTCAGGTGCAGAGAGGAAGG | AGGGGAGTTGAGGATCTCGT |
| *ZmGRF6* | Zm00001d048554_T001 | GTCTGGTGCAGAGAGGAAGG | AGTTGAGAGCAATGCCAAGC |
| *ZmGRF7* | Zm00001d039042_T001 | AATGTGGGAGCAACAGGTTC | AGTTCGTGCGGTCCAATATC |
| *ZmGRF8* | Zm00001d021885_T001 | AAGGTCTTCTACCTCAAGATGAAGG | CAAGGCCAGATCTGCAAGAG |
| *ZmGRF9* | Zm00001d018856_T001 | AATGTGGGAGCAACAGGTTC | CCACCCATATGGACAGTTGA |
| *ZmGRF10* | Zm00001d033357_T001 | GGAGCTCTCTGTGGAGGAGC | CCTCCTCCTTCTGCTCGATG |
| *ZmGRF11* | Zm00001d007968_T001 | CAGCGTCCGCAGGGCCTC | GGATGGGACGAGGTGGGA |
| *ZmGRF12* | Zm00001d017434_T001 | ACTGTGCAACCAATGTGCAATC | GTAGGCGACGGACAGCAGGT |
| *ZmGRF13* | Zm00001d007446_T003 | GAGCTAGACAGCCTGGGAGA | AGCAGCATCCTTGATCTCGT |
| *ZmGRF14* | Zm00001d034593_T001 | TAGCTGGTTGCTTGGGGTAG | GAGGTTCCGCTCCACAAATA |
| *ZmGRF15* | Zm00001d013429_T001 | GAGCTCACTGGAGGAGCACT | GGAAGCAAAGAGCTTGAGGA |
| *ZmGRF16* | Zm00001d006462_T001 | AAGCATGTTCCCCTTCTCCT | CTTGTACTGGTCCCGCATTT |
| *ZmGRF17* | Zm00001d052698_T001 | TCCCATAAGGCTTGGACTTG | GGAATCCTCACCAAGCATGT |
| *ZmGRF18* | Zm00001d031688_T001 | CCACACCACCCCTCGTCTCC | ACATTATCCTCCCGCGACAT |
| *ZmGRF19* | Zm00001d036226_T001 | TCAATTAGATACTCATTTCCCTTCC | AGAAATAGAGTTACTGCAATAGCGC |
| *ZmGRF20* | Zm00001d038649_T002 | GATATTACCTCATCAGAGTTTTTTTCT | CAAATAAGAGGTTCCGCTCC |
| *ZmGRF21* | Zm00001d032231_T001 | CCTACAAGGCTGCTCAGGAC | TCCCCAAGGGTGTCTAACTG |
| *ZmGRF22* | Zm00001d050375_T001 | AGCTGCTTGACTCCCACCTA | CCTTCCTCTCAGCACCAGTC |
| *ZmGRF23* | Zm00001d025617_T001 | AGAGAAAGGACGCTGCTGAG | CGATCGGGTGAGTTGAGAAT |
| *ZmGRF24* | Zm00001d003401_T003 | TGCTTGAGACCCATCTTGTG | TTGTATGCCACCATCGTGTT |
| *ZmGRF25* | Zm00001d050903_T001 | TTGTCATGGAACCCAATGTG | AGCTGTTAATGAGGCGGAAA |
| *ZmGRF26* | Zm00001d035316_T001 | CGGCCATGAAAGGTGACTAT | ACAACCATGGCATGCTGATA |
| *ZmGRF27* | Zm00001d036341_T001 | GGACGTGGATATGAGTGCTG | TGACGATCAGATGTGACCTTTT |
| *ZmGRF28* | Zm00001d048868_T002 | CATCCGGTCCCAAAAGTAGA | CAGGTTAACGAAACCGTCGT |
| *ZmTUB* | Zm00001d009780 | GCTATCCTGTGATCTGCCCTGA | CGCCAAACTTAATAACCCAGTA |
| *ZmGADPH* | Zm00001d049641 | CTGGTTTCTACCGACTTCCTTG | CGGCATACACAAGCAGCAAC |

**Supplementary Table 5.** Motifs of sequences

| **Motif** | **Sequence** |
| --- | --- |
| motif 1 | ALAELAPTHPIRLGLALNFSVFYYEILNSPDRACNLAKQAFD |
| motif 2 | ALIKEYRGKIEAELSKICDGILKLLDSHLVPSATAAESKVFYLKMKGDYH |
| motif 3 | TVEERNLLSVAYKNVIGARRASWRIISSIEQKEEGRGNEAHV |
| motif 4 | EAISELDTLGEESYKDSTLIMQLLRDNLTLWTSDLTEDGGD |
| motif 5 | RYLAEFKTGAERKDAAESTMVAYKAAQDI |
| motif 6 | REENVYMAKLAEQAERYEEMVEFMEKVAK |
| motif 7 | MDVDMSGEERHLFSVGFKNTIGAKRASWR |
| motif 8 | EIKEASKGDAG |
| motif 9 | MCNRSIKKSRAYSLISSPKSG |
| motif 10 | TVDVEE |
| motif 11 | FYPPFVICFHFETLC |
| motif 12 | PTYHETFAYAQGSFARVPEAYVLSLGIYLLPNRECTRTLFTCGIPTIKMW |
| motif 13 | MRDQYRIIYMYRNIQ |
| motif 14 | MESAHKIGGGSVPGAGAGGEGILCHACGYQYPNGHPSAKQR |
| motif 15 | NSGEGVYKDSTLMMQLLKDNLALWTSELT |

**Supplementary Table 6.** The annotation of all predicted genes

| **Maize *14-3-3s*** | **Predicted gene** | **Annotations (Phytozome database)** |
| --- | --- | --- |
| *ZmGRF3* | Zm00001d039694 | LOB transcription factor |
|  | Zm00001d017609 | MYB DNA-binding domain superfamily protein |
|  | Zm00001d043205 | Ethylene-responsive transcription factor |
|  | Zm00001d017788 | DOF zinc finger protein |
|  | Zm00001d033353 | AP2/EREBP transcription factor superfamily protein; Ethylene-responsive transcription factor |
| *ZmGRF4* | Zm00001d033353 | AP2/EREBP transcription factor superfamily protein; Ethylene-responsive transcription factor |
|  | Zm00001d048991 | AP2/EREBP transcription factor superfamily |
|  | Zm00001d039694 | LOB transcription factor |
|  | Zm00001d028524 | Ethylene-responsive transcription factor; AP2/EREBP transcription factor superfamily protein |
|  | Zm00001d052087 | Ethylene-responsive transcription factor; AP2/EREBP transcription factor superfamily protein |
|  | Zm00001d017466 | AP2/EREBP transcription factor superfamily protein; Ethylene-responsive transcription factor |
|  | Zm00001d010676 | Ethylene-responsive transcription factor; AP2/EREBP transcription factor superfamily protein |
|  | Zm00001d015639 | Ethylene-responsive transcription factor; AP2/EREBP transcription factor superfamily protein |
|  | Zm00001d030023 | NAC-transcription factor |
|  | Zm00001d052026 | Ethylene-responsive transcription factor; AP2/EREBP transcription factor superfamily protein |
|  | Zm00001d039324 | Pathogenesis-related genes transcriptional activator PTI6 |
|  | Zm00001d039506 | NAC-type transcription factor |
|  | Zm00001d048474 | MADS transcription factor |
|  | Zm00001d043205 | Ethylene-responsive transcription factor |
|  | Zm00001d034920 | AP2-EREBP transcription factor; Ethylene-responsive transcription factor |
| *ZmGRF6* | Zm00001d039324 | Pathogenesis-related genes transcriptional activator PTI6 |
|  | Zm00001d035514 | Transcription factor IIIA |
|  | Zm00001d042305 | TCP transcription factor |
|  | Zm00001d017268 | MYB transcription factor |
|  | Zm00001d014286 | Calmodulin-binding transcription activator |
|  | Zm00001d045477 | BBR/BPC transcription factor |
|  | Zm00001d049800 | GAGA-binding protein |
|  | Zm00001d048474 | MADS transcription factor |
|  | Zm00001d021537 | MYB transcription factor |
|  | Zm00001d051520 | MYB-related protein Zm1 |
| *ZmGRF8* | Zm00001d042305 | TCP transcription factor |
|  | Zm00001d025720 | Trihelix transcription factor |
|  | Zm00001d017268 | MYB transcription factor |
|  | Zm00001d021927 | BES1/BZR1 protein |
|  | Zm00001d039324 | Pathogenesis-related genes transcriptional activator PTI6 |
|  | Zm00001d048474 | MADS transcription factor |
|  | Zm00001d014286 | Calmodulin-binding transcription activator |
|  | Zm00001d046305 | BES transcription factor |
|  | Zm00001d051520 | MYB-related protein Zm1 |
| *ZmGRF9* | Zm00001d049800 | GAGA-binding protein |
|  | Zm00001d035514 | Transcription factor IIIA |
|  | Zm00001d042305 | TCP transcription factor |
|  | Zm00001d017268 | MYB transcription factor |
|  | Zm00001d045477 | BBR/BPC transcription factor |
|  | Zm00001d014286 | Calmodulin-binding transcription activator |
|  | Zm00001d013849 | Trihelix transcription factor GT-2 |
|  | Zm00001d051520 | MYB-related protein Zm1 |
| *ZmGRF13* | Zm00001d052102 | AP2/EREBP transcription factor superfamily protein |
| *ZmGRF16* | Zm00001d052229 | bZIP transcription factor superfamily protein |
|  | Zm00001d053988 | bZIP transcription factor superfamily protein |
|  | Zm00001d042910 | MYB DNA-binding domain superfamily protein |
|  | Zm00001d001837 | MYB-type transcription factor |
|  | Zm00001d015153 | BZIP transcription factor |
| *ZmGRF18* | Zm00001d030617 | Ocs element-binding factor; bZIP transcription factor superfamily protein |
|  | Zm00001d006053 | NAC domain transcription factor superfamily protein |
|  | Zm00001d034457 | Opaque2 heterodimerizing protein |
|  | Zm00001d043921 | NAC domain transcription factor superfamily protein |
|  | Zm00001d006687 | Homeobox-leucine zipper protein |
|  | Zm00001d017788 | DOF zinc finger protein |
|  | Zm00001d027395 | NAC domain transcription factor superfamily protein |
| *ZmGRF24* | Zm00001d011985 | TCP transcription factor |
|  | Zm00001d033267 | Transcription factor phytochrome interacting factor |
|  |  |  |
| *ZmGRF25* | Zm00001d010309 | SBP-transcription factor |
|  | Zm00001d052087 | Ethylene-responsive transcription factor; AP2/EREBP transcription factor superfamily protein |
|  | Zm00001d017466 | AP2/EREBP transcription factor superfamily protein; Ethylene-responsive transcription factor |
|  | Zm00001d010676 | Ethylene-responsive transcription factor; AP2/EREBP transcription factor superfamily protein |
|  | Zm00001d051355 | Ethylene-responsive transcription factor; AP2/EREBP transcription factor superfamily protein |
|  | Zm00001d015639 | Ethylene-responsive transcription factor; AP2/EREBP transcription factor superfamily protein |
|  | Zm00001d052026 | Ethylene-responsive transcription factor; AP2/EREBP transcription factor superfamily protein |
|  | Zm00001d005892 | Ethylene-responsive transcription factor |
|  | Zm00001d039324 | Pathogenesis-related genes transcriptional activator |
|  | Zm00001d043205 | Ethylene-responsive transcription factor |
| *ZmGRF26* | Zm00001d052026 | Ethylene-responsive transcription factor; AP2/EREBP transcription factor superfamily protein |
|  | Zm00001d048991 | AP2/EREBP transcription factor superfamily |
|  | Zm00001d039694 | LOB transcription factor |
|  | Zm00001d028524 | Ethylene-responsive transcription factor; AP2/EREBP transcription factor superfamily protein |
|  | Zm00001d052087 | Ethylene-responsive transcription factor; AP2/EREBP transcription factor superfamily protein |
|  | Zm00001d017466 | AP2/EREBP transcription factor superfamily protein; Ethylene-responsive transcription factor |
|  | Zm00001d015639 | Ethylene-responsive transcription factor; AP2/EREBP transcription factor superfamily protein |
|  | Zm00001d005892 | Ethylene-responsive transcription factor |
|  | Zm00001d039324 | Pathogenesis-related genes transcriptional activator PTI6 |
|  | Zm00001d043205 | Ethylene-responsive transcription factor |
|  | Zm00001d034920 | AP2-EREBP transcription factor; Ethylene-responsive transcription factor |
| *ZmGRF27* | Zm00001d014286 | Calmodulin-binding transcription activator |
|  | Zm00001d021573 | SBP-transcription factor |
| *ZmGRF28* | Zm00001d008882 | MADS transcription factor |
|  | Zm00001d035514 | Transcription factor IIIA |
|  | Zm00001d021442 | Plant regulator RWP-RK family protein; low quality protein: protein NLP1 |
|  | Zm00001d042618 | MADS-box transcription factor family protein MADS16 |
|  | Zm00001d006094 | MADS27 |
|  | Zm00001d048474 | MADS transcription factor |
